# Predictors of clinical and surgical characteristics of giant stones of the urinary bladder: a retrospective study

**DOI:** 10.1186/s12894-023-01261-2

**Published:** 2023-05-04

**Authors:** Rabea Ahmed Gadelkareem, Mahmoud Mohamad Shalaby, Amr Abou Faddan

**Affiliations:** Assiut Urology and Nephrology Hospital, Faculty of Medicine, Assiut University, Elgamaa Street, Assiut, 71515 Egypt

**Keywords:** Farmers, Lithiasis, Lower urinary tract symptoms, Urinary bladder, Urinary bladder calculi

## Abstract

**Background:**

Giant stones of the urinary bladder (GSBs) are rare and usually presented as case reports. We aimed to assess the clinical and surgical characteristics of GSBs and identify their predictors.

**Methods:**

A retrospective study of 74 patients with GSBs who presented between July, 2005 and June, 2020 was performed. Patients’ demographics, clinical presentations, and surgical peculiarities were studied.

**Results:**

Older age and male gender were risk factors for the occurrence of GSBs. The irritative lower urinary tract symptoms (iLUTS) were the main presenting symptoms (97.3%). Most patients were treated with cystolithotomy (90.1%). Univariate analyses showed that solitary (p < 0.001) and rough surface (P = 0.009) stones were significant factors for occurrence of iLUTS as the presenting symptoms. Also, the severity of symptoms (p = 0.021), rough surface (p = 0.010) and size (p < 0.001) of stones, and farmer occupation (p = 0.009) were significantly associated with adherence of the stone to the bladder mucosa at surgery. In multivariate analysis, the rough surface (p = 0.014) and solitary (p = 0.006) stones, and concomitant ureteral stones (p = 0.020) were independently associated with iLUTS as the main presentation. However, the stone size and severity of iLUTS were the independently associated factors for adherence of GSBs to the bladder mucosa.

**Conclusions:**

Solitary GSB, rough surface and the association with ureteral stones are independent risk factors for the occurrence of long-standing iLUTS. The stone size and severity of iLUTS were the independent predictors of adherence of GSBs to the bladder mucosa. Cystolithotomy is the main treatment, but it may be more difficult when there is bladder mucosa adherence.

## Background

Our region is located within the geographical distribution of the Afro-Asian stone-forming belt, where there is a high prevalence of urolithiasis [1]. Stones of the urinary bladder represent 5% of all urinary stones and usually present with irritative lower urinary tract symptoms (iLUTS). However, there may be no symptoms or minimal iLUTS in a few instances [2, 3]. Owing to the relatively capacious bladder cavity, bladder stones gain variable sizes up to > 20 cm [4]. The giant stone of the urinary bladder (GSB) is defined as a stone which weighs more than 100 gm or measures > 4 cm in its largest dimension [5]. GSBs are rare and have usually been published as individual case reports [6, 7]. They can result in significant morbidities, varying from the relatively common iLUTS to the life-threatening sequels such as malignancy of the urinary bladder [2, 8, 9]. We believe that the level of evidence of results from case series would be higher than that from the individual case reports. Hence, the aim of this study was to assess the clinical and surgical characteristics of GSBs and identify their predicting factors.

## Methods

A retrospective study was carried out by searching the manual and electronic patients’ records of the cases of GSBs that were treated between July, 2005 and June 2020 in our hospital. We defined GSB as a stone with a size ≥ 4 cm and located within the proper cavity of the urinary bladder, bladder diverticulum, or in a neobladder. Each case was reviewed for demographics including age, gender, occupation, and residence. Regarding clinical presentations, iLUTS were evaluated according to the American Urological Association Symptom Index for benign prostatic hyperplasia (BPH), before and after surgery [10]. A validated Arabic version of this tool was used in most of cases, as it was not available in the early years of the study [11]. Other clinical characteristics included a history of previous surgery, imaging-based characteristics (type of imaging, upper urinary tract stones, and topographic features of the stones, including number, size, outline or surface, and shape), complications, and lines of treatment were also recorded. The primary outcome of this study was the incidence of iLUTS as the main presenting symptoms. The secondary outcome was the presence of stone adherence to the bladder mucosa at surgery. Accordingly, the possible risk factors were studied. Follow-up outcomes were evaluated throughout the first year after surgery.

### Statistical analysis

The statistical package for social sciences, version 20.0 (SPSS Inc., Chicago, Illinois, USA) was used to analyze the data. In descriptive analyses, continuous variables were presented as mean ± standard deviation (SD) and range. However, categorical variables were presented as the number and percentage of each category. Two-tailed P < 0.05 was considered as statistically significant.

## Results

Out of more than 82,000 urological procedures that were performed during the period of the study, only 74 cases (0.1%) were operated upon for GSBs. The demographic and clinical characteristics are presented in Table 1.

**Table 1:** Patients’ demographic and clinical characteristics (n = 74)

Characteristics	Mean ± SD or Number (Percentage)
Age (yr)	59 ± 14
Gender	
Male	69 (93.2)
Female	5 (6.8)
Body mass index (kg/m^2^)	24.8 ± 5.5
Residence	
Rural	67 (90.5)
Urban	7 (9.5)
Education level	
High	4 (5.4)
Medium	6 (8.1)
Low	28 (37.8)
None	36 (48.6)
Occupation	
Farmer	56 (75.7)
Non-farmer	18 (24.1)
Major comorbidity	
Diabetes mellitus	13 (17.6)
Cardiovascular/Hypertension	22 (29.7)
Chronic kidney disease	6 (8.1)
Main presenting symptom	
Accidental discovery	2 (2.7)
iLUTS	66 (89.2)
iLUTS plus hematuria	6 (8.1)
Degree of symptoms	
Mild	12 (16.2)
Moderate	25 (33.8)
Sever	36 (48.6)
Not graded	1 (1.4)
Duration of symptoms (ms)	20.1 ± 26.4
Category of underlying etiology	
Bladder pathology	16 (21.6)
Infra-vesical obstruction	43 (58.1)
Unknown	15 (20.3)
Urine reaction	
Acidic	26 (35.1)
Alkaline	48 (64.9)
Culture and sensitivity results	
Negative	26 (35.1)
Positive	45 (60.8)
Major complications	
Bladder cancer	3 (4.1)
Hematuria	2 (2.7)
Inguinal hernia	2 (2.7)
Rectal prolapse	2 (2.7)
Urge incontinence.	2 (2.7)
Urge incontinence plus UTI	2 (2.7)
UTI	45 (60.8)
Imaging	
US, KUB	47 (63.5)
US, KUB, AUG	2 (2.7)
US, KUB, IVU	7 (9.5)
US, KUB, MSCT	14 (18.9)
US, MSCT	4 (5.4)
Features of stones in imaging	
Stone size (cm)	5.8 ± 1.6
Stone number	
Single	66 (89.2)
Multiple	8 (10.8)
Stone shape	
Rounded	70 (94.6)
Oval	4 (5.4)
Stone surface	
Smooth	22 (29.7)
Serrated	48 (64.9)
Spiky	4 (5.4)
Associated upper urinary tract stones	
Stone kidney	6 (8.1)
Stone ureter	15 (20.3)
None	53 (71.6)
Lines of treatment of stones	
Radical cystectomy	3 (4.1)
Cystolithotomy plus upper tract interventions ^a^	12 (16.2)
Cystolithotomy plus surveillance ^b^	4 (5.4)
Cystolithotomy only	51 (68.9)
Endoscopic lithotripsy	4 (5.4)
Stone surface at operation	
Smooth	10 (13.5)
Rough	60 (81.1)
Spiky	4 (5.4)
Stone adherence to bladder mucosa	
Adherent	21 (28.4)
Non-adherent	53 (71.6)
Outcomes of 1-year Follow-up after Treatment	
Death	4 (5.4)
Stone recurrence	1 (1.4)
Resolution of the symptoms ^c^	21 (28.4)
Persistence of symptoms:	48 (64.9)
Managed by medications for BPH	12 (16.2)
Managed by medications for neurogenic bladder	10 (13.5)
Needed TURP or TVP ^d^	15 (20.3)
Needed visual internal urethrotomy ^d^	3 (4.1)
Urethroplasty	2 (2.7)
Fulguration of posterior urethral valve	1 (1.4)
Regular bladder neck dilatation	1 (1.4)
Others, including patients who refused further interventions	4 (5.4)

*AUG* ascending urethrocystography, *IVU* intravenous urography, *KUB* kidney-ureter-bladder radiography, *iLUTS* irritative lower urinary tract symptoms, *MSCT* multislice computed tomography, *PNL* percutaneous nephrolithotomy, *SD* standard deviation, *TURP* transurethral resection of the prostate, *TVP* transvesical prostatectomy, *US* ultrasonography, *UTI* urinary tract infection

^a^Upper tract interventions included PNL in 2 cases and ureteroscopy in 10 patients

^b^Surveillance was performed in 4 elderly patients with non-obstructing calyceal kidney stones

^c^Resolution of symptoms refers to complete disappearance or significant reduction of iLUTS. They included 15 patients with stone adherence to the bladder mucosa. Non-resolution or persistence of symptoms was defined as continuation of iLUTS in a lower rate or degree and/or appearance or progression of other obstructive symptoms due to unmask effect after resolution of iLUTS.

^d^These procedures were performed simultaneous to the surgery for the bladder stones in 5 cases of TURP, the single case of TVP and 3 cases of visual internal urethrotomy

iLUTS was the main presenting symptoms in 72 patients (97.3%). Of them, only 6 patients (8.1%) presented with hematuria as a main symptom plus iLUTS. Only, 2 GSBs were accidentally discovered within neobladders (2.7%) during their follow-up. Duration of symptoms ranged from 3 months to 10 years.

**Fig. 1:**
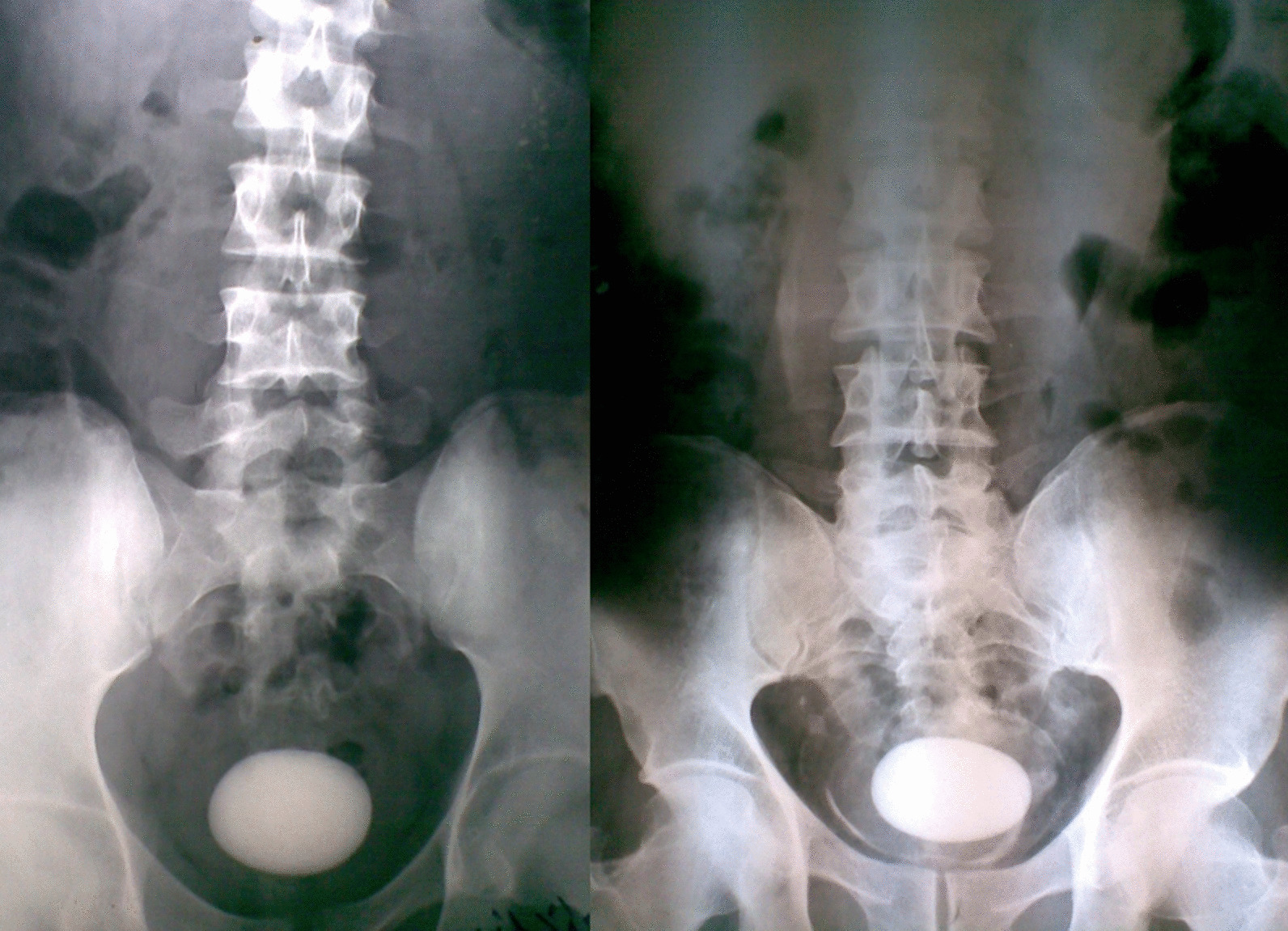
Giant stones of the urinary bladder with oval shapes, smooth surfaces, and high densities in plain radiographs. Notice the linear bilharzial calcifications of the urinary bladder in the right-sided figure

**Fig. 2:**
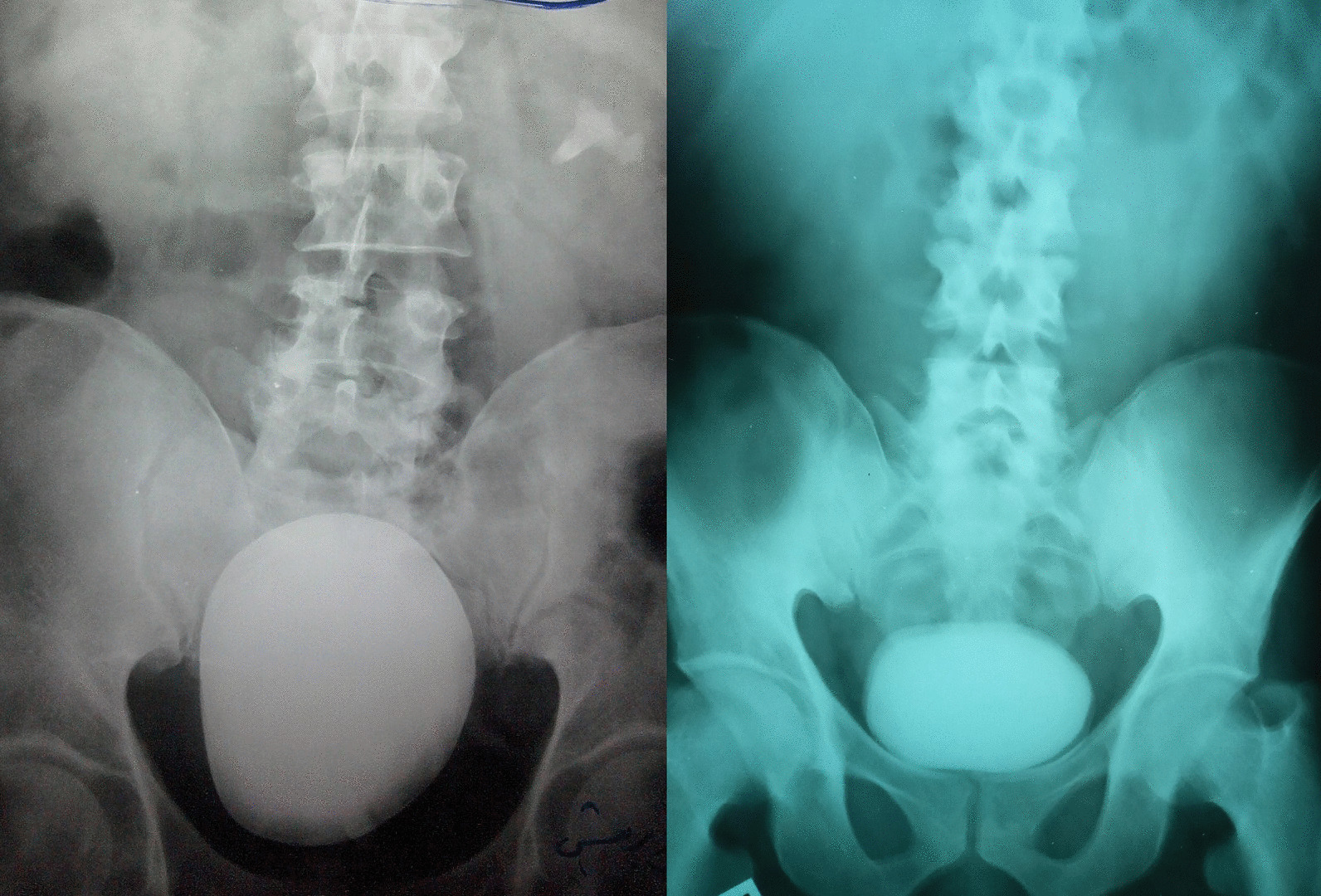
Single huge oval stones with high densities and different lies in plain radiographs: The left-sided figure shows is a huge stone with a vertical lie in a patient with a neobladder and a co-existent branched left kidney stone. The right-sided figure shows a huge stone with a transverse lie and a faint layer on a huge dense core in a native bladder

**Fig. 3:**
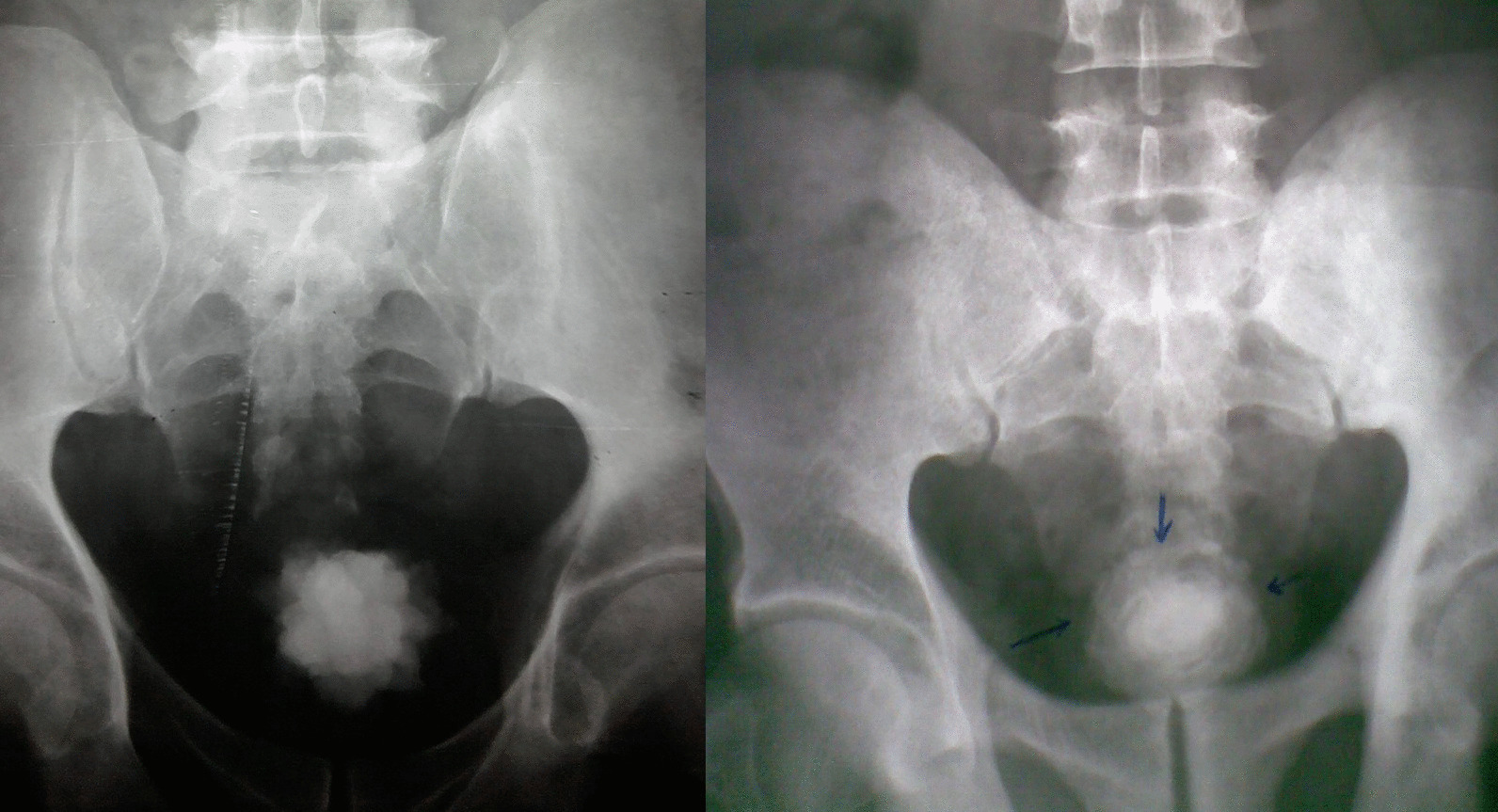
Giant stones of the urinary bladder with rounded shapes, rough surfaces, and moderate densities in plain radiographs: The left-sided one has a prominently serrated surface (a spiky stone) and the right- sided one has a less-prominently serrated surface

**Fig. 4:**
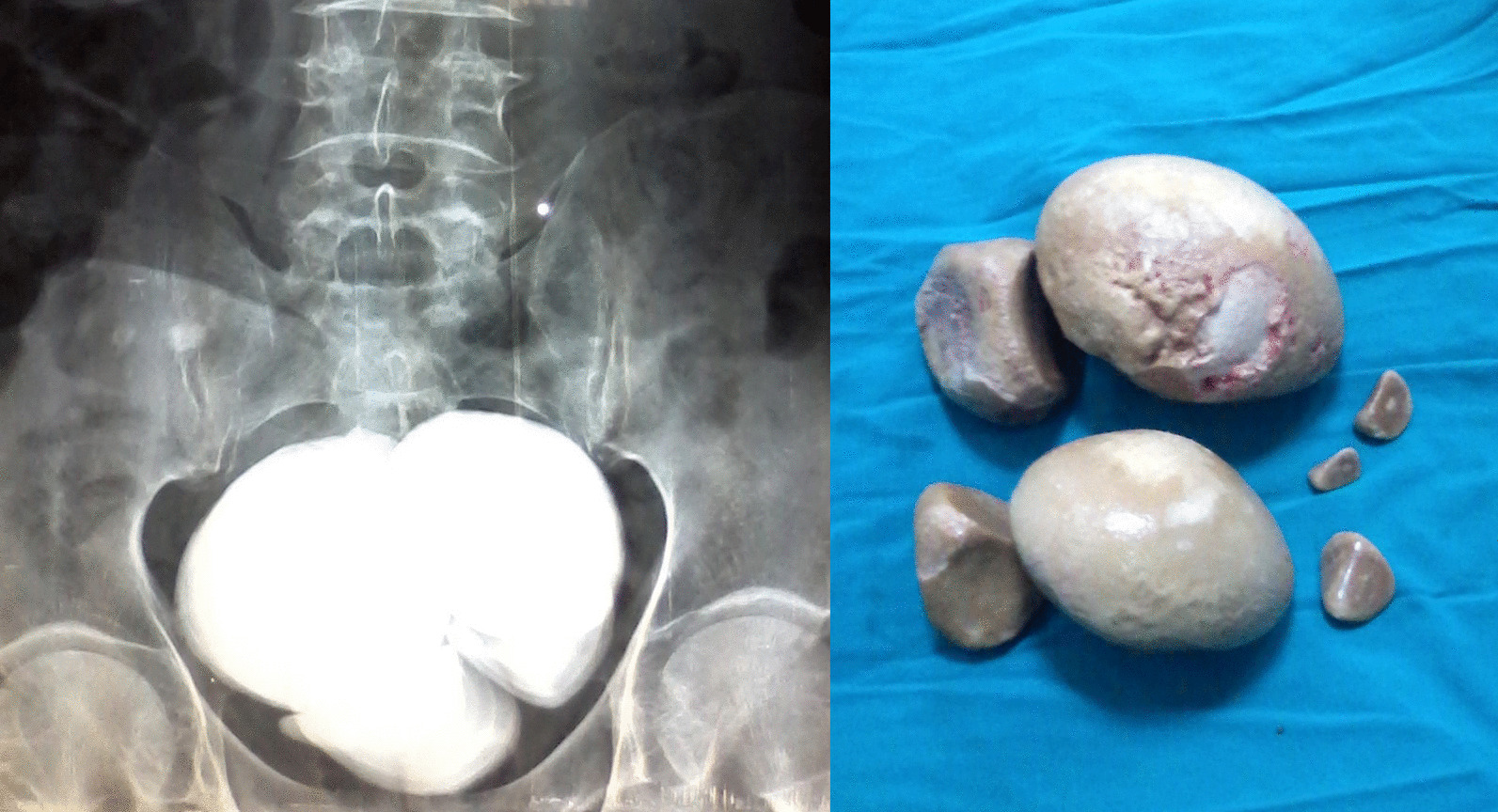
Multiple giant stones of the urinary bladder in a patient with neurogenic voiding dysfunctions: The left-sided figure is a plain radiography showing dense overlapping bladder stones. The right-sided figure shows the gross appearance of these stones after cystolithotomy

The stones were detected by abdominal ultrasonography as an initial imaging in all cases (100%). Also, the plain kidney-ureter-bladder radiograph was a basic imaging and showed peculiar topographic characteristics, regarding the stone size, surface, and radio-opacity (Tables 1 and 2; Figs. 1, 2, 3 and 4). Further imaging was directed towards the underlying etiology or the upper urinary tract stones, and included intravenous urography and multi-slice computed tomography (Table 1).

**Table 2:** Univariate analysis of the risk factors of the etiology of the giant stones of the urinary bladder (One-way ANOVA for the continuous variables and Fisher’s Exact test for the categorical variables)

Variables	Bladder pathology	Infravesical obstruction	Unknown causes	*p* value
	Mean ± standard deviation or number (percentage)			
Age (years)	52.4 ± 17.3	61.5 ± 12.9	58.9 ± 12	0.038
Gender				
Male	14 (18.9%)	43 (58.1)	12 (16.2%)	0.017
Female	2 (2.7%)	0	3 (4.1%)	
Residence				
Rural	14 (18.9%)	41 (55.4%)	12 (16.2%)	0.194
Urban	2 (2.7%)	2 (2.7%)	3 (4.1%)	
Education level				
High	2 (2.7%)	2 (2.7%)	0	0.526
Middle	0	4 (5.4%)	2 (2.7%)	
Low	14 (19%)	37 (50%)	13 (17.6%)	
Occupation				
Farmer	12 (16.2%)	35 (47.3%)	9 (12.2%)	0.250
Non-farmer	4 (5.4%)	8 (10.8%)	6 (8.1%)	
Urinary pH				
Acidic	6 (8.3%)	17 (23.6%)	3 (4.2%)	0.336
Alkaline	8 (11.1%)	26 (36.1%)	12 (16.7%)	
Urine culture and sensitivity				
Negative	6 (8.5%)	17 (23.9%)	3 (4.2%)	0.318
Positive	8 (11.3%)	25 (35.2)	12 (16.9%)	

The underlying etiology was categorized into bladder pathology (21.6%), infravesical obstruction (58.1%), and unknown etiology (20.3%). Bladder pathology included neurogenic bladder in 14 patients (18.9%) and neobladder in 2 patients (2.7%). Infravesical obstruction included BPH in 36 patients (48.6%), bladder neck contracture in 1 patients (1.4%), urethral stricture in 5 patients (6.8%), and neglected posterior urethral valve in 1 patient (1.4%).

The causes of delayed presentation of patients included fear of surgery in 24 patients (32.4%), attributing the symptoms to prostatic enlargement in 28 patients (37.8%), absence of nearby health facilities in 13 patients (17.6%), and undetermined causes in 9 patients (12.2%).

As a routine step before endoscopic lithotripsy or cystolithotomy, cystoscopy was performed in all patients (100%) for exclusion of any concomitant malignancy. Associated bladder cancer was diagnosed in 3 patients (4.1%) and confirmed by a transurethral resection biopsy. The pathological type was squamous cell carcinoma in 2 patients and transitional cell carcinoma with squamous differentiation in one patient. All the three cases were treated by radical cystectomy and urinary diversion.

Sixty-seven patients (90.5%) were treated by cystolithotomy, either via a lower abdominal midline incision in 54 patients (80.6%) or via a Pfannenstiel incision in 13 patients (19.4%). The stones were adherent to the bladder mucosa in 21 patients (31.3%) with relatively very large stones, warranting a large vertical cystotomy and gradual delivery in these patients. In detail, some maneuvers were used to facilitate stone delivery of the adherent stones and included transrectal digital assisted delivery of the stone in 8 patients, excision of interdigitating mucosal patches with the stone surface in 3 patients and counter-traction of the bladder wall edges with stone forceps-assisted mobilization of the stone in all 21 patients. Unplanned bladder tear occurred in 3 patients and was meticulously repaired without a need for further interventions. No bladder perforations were encountered, hence we did not perform confirmatory postoperative cystograms. Prophylactically, a final check for urinary leak was performed before closure of the bladder in two-layer water-tight fashion, using continuous absorpable sutures. In addition, a retropubic drain was routinely placed in all cases. Furthermore, the duration of urethral catheter was prolonged to ≥ 7 days in 17 patients with adherent stones and in all patients with neurogenic bladder or neobladders.

Four patients (5.4%) refused cystolithotomy and were treated with a transurethral endoscopic lithotripsy, using pneumatic lithotripters. In one of them (1.4%), a two-session transurethral lithotripsy was combined with extracorporeal shockwave lithotripsy, which facilitated the clearance of the stone fragments during the second session.

Stone analysis was found in the records of 19 cases (25.7%) only. All the analyzed stones were mixed stones with predominance of the calcium oxalate crystals. Resolution of symptoms occurred in 21 patients (28.1%), including 16 patients from those patients with GSBs adherence to the bladder mucosa. However, there were 48 patients (64.9%) with persistent symptoms and they were treated by medications (29.7%) or surgical interventions (29.7%) for the underlying pathology. Four patients (5.4%) died within 1-year follow-up. Only one patient (1.4%) died within 30 days postoperatively due to deep venous thrombosis and pulmonary embolism, but the other three patients died after 30 days, due to cerebral strokes in two patients (2.7%) and myocardial infarction in one patient (1.4%) (Table 1).

Univariate analyses showed that older age and male gender were risk factors for the occurrence of GSBs (Table 2). Also, solitary stone, rough surface of these stones and association with ureteral stones were significant factors for occurrence of iLUTS as the main presenting symptoms (Table 3). The severity of these symptoms, stone size and surface, and the patient’s occupation as a farmer were significantly associated with adherence of the stone to the bladder mucosa at surgery (Table 4).

**Table 3:** Univariate analysis of the risk factors of occurrence of iLUTS as the main presenting symptoms in patients with giant bladder stones (Fisher’s Exact test for categorical variables and One-way ANOVA for continuous variables)

Variables	Accidental	iLUTS	iLUTS with hematuria	*p* value
	Percentage of total number of patients (% of 74) or Mean ± SD			
Stone number				
Single	1.4	85.1	2.7	< 0.001
Multiple	0	5.4	5.4	
Stone surface				
Smooth	1.4	28.4	0	0.009
Rough	0	59.5	5.4	
Spiky	0	2.7	2.7	
Association with ureteral stones				
Present	1.4	16.2	4.1	0.002
None	0	74.3	4.1	
Age (year)	7.7	63	3	0.806
Stone size (cm)	5.8 ± 1.7	59.2 ± 14.3	21 ± 27.3	0.438
Duration of the diseases (month)	5.4 ± 1.6	55.7 ± 12.7	9.3 ± 2.1	0.559

*iLUTS* irritative lower urinary tract symptoms, *SD* standard deviation

**Table 4:** Univariate analysis of the risk factors for presence of adherence of the stone to the bladder mucosa at surgery (Fisher’s Exact test for categorical variables and Independent t test for continuous variables)

Variables	Adherence to mucosa	None	*p* value
	Mean ± standard deviation or percentage of total number of patients (% of 74)		
Age (year)	61.9 ± 13	57.95 ± 14.4	0.499
Stone size (cm)	7.5 ± 1.9	5.1 ± 0.82	< 0.001
Duration of symptoms (month)	17.3 ± 21.1	21.2 ± 28.2	0.431
Degree of symptoms			
Mild	5.4	10.8	0.021
Moderate	2.7	31.1	
Sever	2.7	29.7	
Not assessed	1.4	0	
Occupation			
Farmer	27	48.6	0.010
Non-farmer	1.4	23	
Stone surface			
Smooth	1.4	28.4	0.009
Serrated	0	59.5	
Spiky	0	2.7	

Multivariate analyses showed that the association with ureteral stones, rough stone surface and solitary stones were independent risk factors for the occurrence of iLUTS as the main presenting symptoms in patients with GSBs. The stone size and severe degree of iLUTS were the only independent predictors of adherence of the stone to the bladder mucosa (Table 5).

**Table 5:** Multivariate logistic regression analyses of variables that may affect the occurrence of iLUTS as the main presenting symptoms and adherence of the stone to the bladder mucosa

Variables	B	S.E.	Wald	95% CI	*p* value
iLUTS as the presenting symptoms					
Stone number	6.432	2.318	7.701	6.613–58351.1	0.006
Stone surfaces	− 1.834	1.243	2.175	0.014–1.828	0.014
Association with ureteral stones	− 3.445	1.485	5.379	0.002–0.587	0.020
Adherence of GSB to the bladder mucosa					
Stone size	− 4.340	1.534	8.006	0.001–0.264	0.005
Degree of iLUTS	16.040	0.762	442.946	2.272–5.638	< 0.001
Farmer occupation	− 2.122	1.090	3.793	0.014–1.014	0.051
Stone surface	0.230	1.433	0.026	0.076–20.884	0.872

Accidentally discovered cases were excluded

*CI* confidence interval, *GSB* giant stones of urinary bladder, *iLUTS* irritative lower urinary tract symptoms

## Discussion

GSBs may represent a different entity from the small stones, in regards to the etiology and treatment [7, 12, 13]. Enormous and extremely large-sized GSBs are few in the literature [5]. Predisposing factors include the infravesical obstruction, neurogenic voiding dysfunctions, UTIs, and neobladders [6, 7, 12–15]. In low-resource regions such as the rural areas, many sociodemographic factors may have a role in the formation of GSBs. Poor diets and water supply are prevalent in these areas and may aggravate the metabolic mechanisms of urolithiasis. Also, unavailability of proper health facilities may delay the diagnosis and proper management of the underlying causes [5, 14]. Similarly, the major proportions of our cases came from rural areas and had low educational levels. All these factors could occur in the farmer occupation, where it showed a significant effect on the occurrence of iLUTS in patients with GSBs. Farmers live in rural areas, where proper health facilities and food and water intake may be unavailable, as mentioned previously. Although this seems to be the cause in the farmer occupation in our locality, it should be taken with caution for those in other localities, due to the variability of these factors among farmers worldwide.

Association between bladder stones and bladder carcinomas should be suspected in patients with long-standing GSBs presenting with hematuria [5]. This association has been reported in a few occasions in the literature [2, 8, 9]. It is attribued to the chronic irritation and squamous metaplasia [2]. However, the causality between GSBs and malignancy in this association is not fully understood, where squamous cell carcinoma and other histological variants have been reported [8]. In our study, there was no direct relationship between the size of the stones and the development of bladder carcinoma. This could be attributed to the very small number of these cases. We encountered both squamous and transitional cell carcinomas. Also, other causes of bladder carcinoma such as schistosomiasis are prevalent in our locality, rendering the identification of the actual underlying cause relatively difficult.

GSB presents commonly as a solitary stone. However, multiple GSBs have been reported in both genders [14, 16]. Multiplicity of GSBs may be associated with certain underlying etiologies such as UTIs [14, 16], neurogenic disorders [15], and intestinal urinary reconstruction [17–19]. In our study also, we have reported patients with multiple GSBs in a neurogenic bladder. This finding may support a proposed etiological role of UTIs in the multiplicity of GSBs, because there is a common association between the urinary stasis and UTIs in patients with bladder outlet obstruction or neurogenic bladder [16, 20, 21].

Although iLUTS is the usual clinical presentation of GSBs and is usually of long duration, they are not pathognomonic to GSBs [5, 14, 22, 23]. The possible causes of this delayed presentation include poverty, ignorance, poor health service-seeking and reliance on treatment by over-the-counter medication [5, 23]. Similarly, many of our cases had a long-standing history of iLUTS before the diagnosis of GSB. The iLUTS can be attributed to stone-related factors, including the foreign body-like effect of the stone, UTIs, and associated underlying pathology such as prostatic enlargement [5, 14, 22]. Our results were similar, where the majority of our patients had UTIs, rough stone surfaces, and infravesical obstruction. In multivariate analysis, the severity of iLUTS was significantly associated with the presence of ureteral stones, rough surface and solitary stones.

The significant association of ureteral stones with the incidence and severity of iLUTS in patients with GSBs can be attributed to the augmentation effect of the co-existance of two pathologies as etiological factors of iLUTS. In addition, the ureteral stones may be associated with ureteral obstruction and urinary stasis which are risk factors of UTI, adding further risks for development of iLUTS [1]. Regarding the significant association of the solitary GSBs with a higher incidence of iLUTS, when compared to the multiple GSBs, we believe that this significat association might be attributed to the common incidence of the single GSBs [5, 7]. In addition, the multiple GSBs are usually associated with detrusor hypoactivity rather than detrusor overactivity, indicating a lower incidence of iLUTS [21, 24]. However, the effect of the stone size on the occurrence of iLUTS in our results was mostly due to the foreign body-like effect, leading to mucosal irritation and inflammation that were described in the previous cases [2, 22].

The adherence of GSBs to the bladder mucosa at surgery was a peculiar finding which may support the irritative effects of these stones. GSBs with a smooth surface are usually not adherent to the bladder mucosa [15]. This renders delivery of the stones with smooth surfaces easier than those with rough surfaces. At surgery, the delivery of rough surface stones may warrant assisted delivery techniques, including the digital rectal manipulation [23]. The rough surface of GSB seemed to be dependent on the effect of stone size, as its effect could not be proven by the multivariate analysis. We found that only the stone size and severity of iLUTS were independent factors for adherence of the stone to the blaader mucosa. During cystolithotomy, this peculiar surgical finding necessitated a significant caution while delivering those huge stones with rough surfaces. It warranted a generous cystotomy and gradual dislodging of these stones from the bladder cavity due to interdigitating mucosa and serrations of stone surfaces.

Generally, there is a controversy whether to perform simultaneous surgeries for BPH and bladder stones. This concern has been raised in some observational case-series, but the lack of randomization and long-term follow-up leave this controversy unresolved [12, 13]. In the current study, the main clinical presentation was iLUTS. Hence, the surgical interventions were carried out in a sequential manner for patients with underlying etiologies necessitating surgical interventions, including those patients with BPH. Accordingly, most of patients had their symptoms resolved after treatment of GSBs or their underlying causes. These underlying etiologies were treated when the iLUTS persisted or new symptoms supervened after the treatment of iLUTS caused by GSBs.

Unusual huge size of GSBs may form a palpable or visible suprapubic mass [4, 5]. Other rare outcomes have been reported, including bladder rupture, intestinal perforation, spontaneous expulsion [25], and anuria [5, 26]. We did not encounter any of these unusual presentations among our patients.

To the best of our knowledge, the current case series of GSBs is the only one in the literature which included such large number of GSBs. There is a big difference between the number of patients in our study (74 patients) and the previously published series of GSBs, where none of them reported more than 10 patients with GSBs [4, 27].

Limitations of this study included the retrospective nature of the study and unavailability of some data, such as the biochemical analysis of the stones in a major proportion of patients. Also, due to the relatively small number of patients in the categories of the underlying etiology the identification of the predictors of GSBs formation in each category by multivariate analyses was not possible. In future, multi-center studies and presentation of the experiences is recommended.

## Conclusions

GSBs are rare clinical findings in urological practice and tend to occur in relatively older males with predisposing factors such as infravesical obstructions and reluctance to seek medical advice. A history of long-standing iLUTS is the main clinical feature, except in patients with neobladders. Solitary stone, rough stone surface and the association with ureteral stones are independent risk factors for the occurrence of long-standing iLUTS. The stones size and severity of iLUTS were the independent predictors of adherence of the GSB to the bladder mucosa. Co-existent bladder malignancy is potentially possible, but rare. Cystolithotomy is the most suitable line of treatment, and may be more difficult when there is bladder mucosa adherence. Endoscopic lithotripsy with or without extracorporeal shockwave lithotripsy can be employed in limited circumstances. We believe that the findings of the current study will clinically help those future patients presenting with GSBs in planning of proper management, including prediction of the risk factors and suitable surgical planning.

## Data Availability

The data used and analyzed during the current study are available from the corresponding author on reasonable request.

## Abbreviations

BPH: Benign prostatic hyperplasia
GSB(s): Giant stone(s) of the urinary bladder
iLUTS: irritative lower urinary tract symptoms
SD: Standard deviation
UTI(s): Urinary tract infection(s)
